# Exploration and prognostic analysis of two types of high-risk ovarian cancers: clear cell vs. serous carcinoma: a population-based study

**DOI:** 10.1186/s13048-024-01435-y

**Published:** 2024-06-01

**Authors:** Tingwei Liu, Yueqing Gao, Shuangdi Li, Shaohua Xu

**Affiliations:** Department of Gynecology, Shanghai First Maternity and Infant Hospital, School of Medicine, Tongji University, Shanghai, China

**Keywords:** Ovarian clear cell carcinoma (OCCC), Advanced stage, Prognostic factors, Liver metastases, Nomogram, SEER

## Abstract

**Background:**

Ovarian clear cell carcinoma (OCCC) is a rare pathological histotype in ovarian cancer, while the survival rate of advanced OCCC (Stage III-IV) is substantially lower than that of the advanced serous ovarian cancer (OSC), which is the most common histotype. The goal of this study was to identify high-risk OCCC by comparing OSC and OCCC, with investigating potential risk and prognosis markers.

**Methods:**

Patients diagnosed with ovarian cancer from 2009 to 2018 were identified from the Surveillance, Epidemiology, and End Results (SEER) Program. Logistic and Cox regression models were used to identify risk and prognostic factors in high-risk OCCC patients. Cancer-specific survival (CSS) and overall survival (OS) were assessed using Kaplan-Meier curves. Furthermore, Cox analysis was employed to build a nomogram model. The performance evaluation results were displayed using the C-index, calibration plots, receiver operating characteristic (ROC) curve, and decision curve analysis (DCA). Immunohistochemically approach was used to identify the expression of the novel target (GPC3).

**Results:**

In the Cox analysis for advanced OCCC, age (45–65 years), tumor numbers (total number of in situ/malignant tumors for patient), T3-stage, bilateral tumors, and liver metastases could be defined as prognostic variables. Nomogram showed good predictive power and clinical practicality. Compared with OSC, liver metastases had a stronger impact on the prognosis of patients with OCCC. T3-stage, positive distant lymph nodes metastases, and lung metastases were risk factors for developing liver metastases. Chemotherapy was an independent prognostic factor for patient with advanced OCCC, but had no effect on CSS in patients with liver metastases (*p* = 0.0656), while surgery was significantly related with better CSS in these patients (*p* < 0.0001) (*p* = 0.0041). GPC3 expression was detected in all tissue sections, and GPC3 staining was predominantly found in the cytoplasm and membranes.

**Conclusion:**

Advanced OCCC and OCCC with liver metastases are two types of high-risk OCCC. The constructed nomogram exhibited a satisfactory survival prediction for patients with advanced OCCC. GPC3 immunohistochemistry is expected to accumulate preclinical evidence to support the inclusion of GPC3 in OCCC targeted therapy.

**Supplementary Information:**

The online version contains supplementary material available at 10.1186/s13048-024-01435-y.

## Introduction

Epithelial ovarian cancer (EOC) is the most lethal gynecologic malignancy, as well as a morphologically and biologically heterogeneous disease [1, 2]. Due to the aggressiveness of ovarian cancer and the lack of specific symptoms for early detection, there are still no effective tools for general population screening. And previous studies about the financial cost associated with early detection and late-stage treatment of ovarian cancer have consistently demonstrated that the treatment expenses for ovarian cancer remains the highest compared to other types of cancers [3], thereby exerting substantial pressure on both society and individuals. The incidence of ovarian clear cell carcinoma (OCCC) as a pathological type of EOC is quite high in Asia, and it is increasing year by year [4–6].

It has been reported that OCCC is strongly associated with endometriosis and ovarian endometrioid carcinoma [7, 8]. In comparison to other subtypes of EOC, OCCC frequently manifests as a large pelvic mass [9], and the majority of OCCC patients are diagnosed at an early stage with an estimated 5-year survival rate of 90% [5, 10]. Furthermore, the median age of onset of OCCC is 55 years, which is younger than that of serous carcinoma. Of note, the response rate to platinum-based therapy of OCCC is extremely low, particularly in the advanced stage, and advanced OCCC (Stage III-IV) has a poorer prognosis and higher recurrence rate than high-grade serous carcinoma (HGSC) [11].

Molecular characteristics of OCCC are greatly different from ovarian serous carcinoma (OSC), ARIDA loss has often been shown in OCCC to functionally cooperate with PI3K/AKT signaling pathway related mutations, such as loss of PTEN, which is uncommon in HGSC [12]. The p53 mutation frequency in HGSOC is as high as 96%. In contrast, p53 mutations are present in less than 20% of advanced OCCC cases [13]. Compared with HGSOC, BRCA mutations were reported with very low frequency in OCCC (6.3%) [14]. Moreover, high-risk OCCC exhibits more aggressive malignant behaviour compared to OSC. However, there is currently no precise therapeutic schedule developed for OCCC. The current management and treatment of OCCC is still based on the standard therapeutic regimen of OSC [15, 16], which accounts for 90% of ovarian cancer. A proper treatment regimen necessitates the support of a large number of future clinical investigations, particularly for advanced stage OCCC, which is more resistant to chemotherapy and more prone to relapse [17]. Moreover, there are differences in invasion tendencies and survival outcomes among the histologic subtypes of EOC. Kurman et al. proposed that ovarian cancer be divided into two types. Type I, which is genetically stable, such as clear cell, low-grade serous, endometrioid, is typically more indolent and presents at an earlier stage, and therefore has better survival outcomes. Genetically unstable Type II, consisting of high-grade serous carcinomas, undifferentiated carcinomas, and carcinosarcomas, behaves in a more aggressive manner, and typically presents at a later stage [12]. Nicholas Pavlidis et al. have also investigated the differential effects of various treatment regimens on different histological types of ovarian cancer [18]. Based on the above theory, OSC and OCCC belong to type II and type I, respectively. Regrettably, the prognostic factors that affect the survival of OCCC patients but not OSC patients have not been extensively investigated. It is imperative for our researchers to become acquainted with the factors which influence the prognosis of women diagnosed with high-risk OCCC.

The present study investigated potential prognostic factors in the survival of advanced OCCC patients. Subsequently, we comprehensively compared the epidemiological characteristics between OCCC and OSC in order to determine the particular prognostic factors of OCCC. In addition, the impact of various treatments on the survival of these high-risk OCCC patients, as well as risk factors for developing liver metastases, were investigated. The accumulation and summarization of clinical characteristics and therapies could facilitate clinical decision making and future precision therapy, and our objective was to contribute to the clinical evidence.

## Results

### Survival rates

**Fig. 1:**
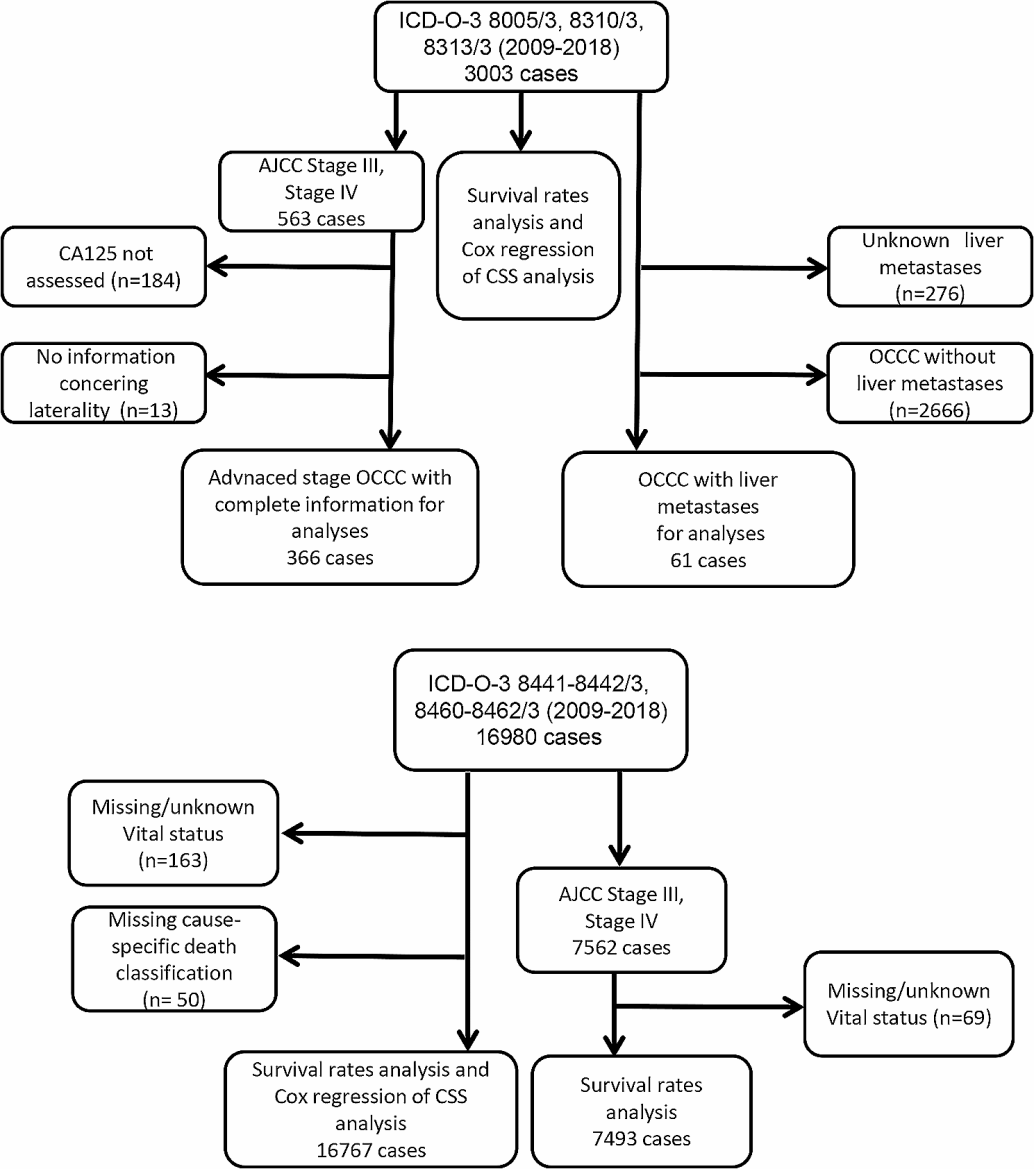
Flowchart of patient selection from the SEER database. SEER, the Surveillance, Epidemiology, and End Results database

**Fig. 2:**
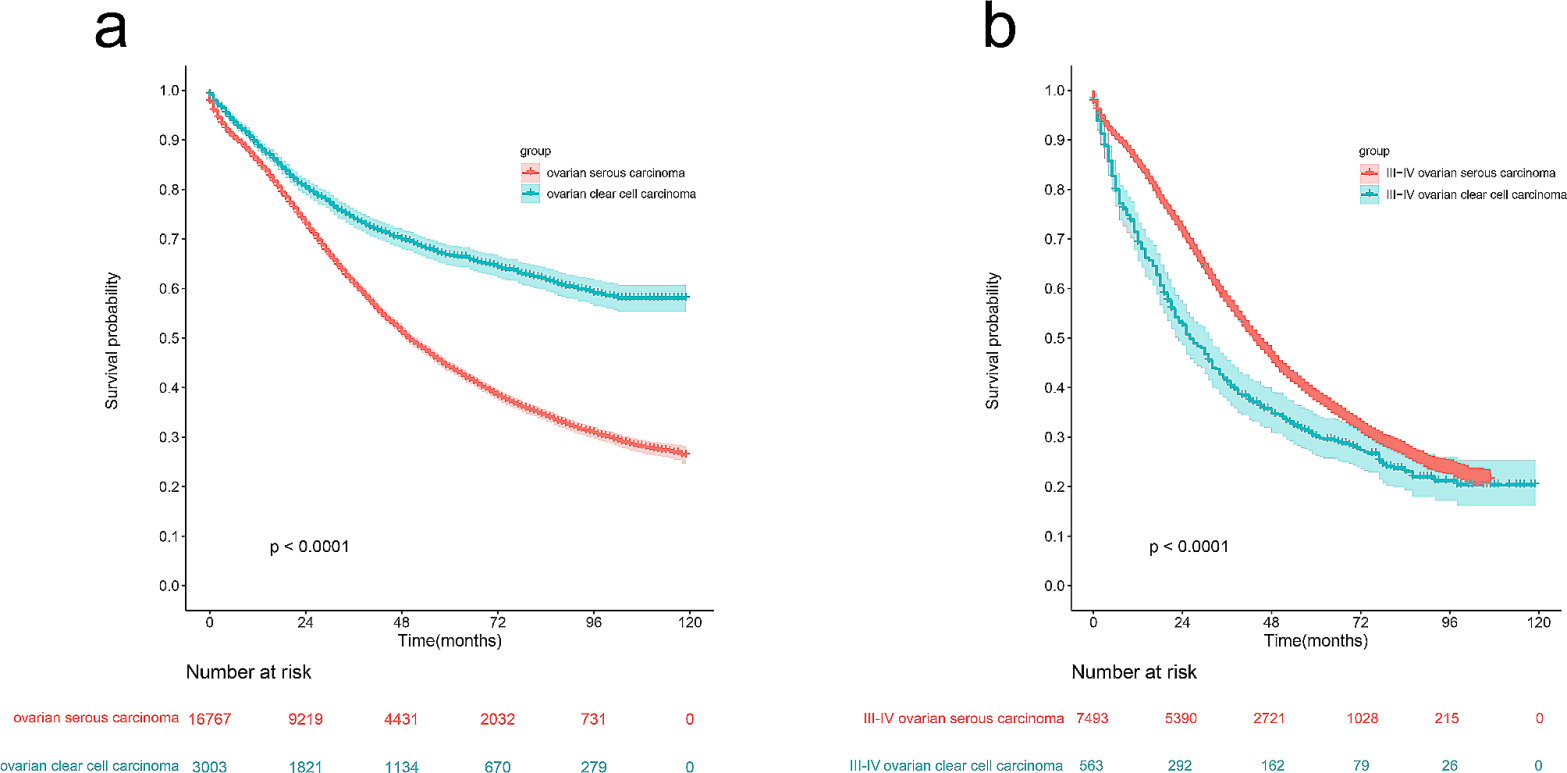
Kaplan–Meier curves for overall survival in OCCC and OSC. (**a**) OSC vs. OCCC; (**b**) stage III-IV OCCC vs. stage III-IV OSC. OSC, ovarian serous cancer; OCCC, ovarian clear cell carcinoma

The flow diagram of participants recruited is shown in Fig. 1. A total of 3003 OCCC cases and 16,767 OSC cases with complete survival information were included in the survival rates analysis. As shown in Fig. 2, OCCC patients had better survival compared with OSC (*p* < 0.001). The median survival time of OCCC patients was 119 (97.34-119.38) months, surpassing the 50.19 (48.60-51.39) months of OSC. This result is consistent with previous literature reports. However, when advanced cases were extracted according to stage, the survival rate of stage III–IV OCCC was significantly lower than stage III–IV OSC (*p* < 0.001). Using the KM method, the overall 1-, 3-, and 5-year survival rates of advanced OCCC were 69.3%, 41.2%, and 30.1%, respectively; the overall 1-, 3-, and 5-year survival rates of advanced OSC were 85.3%, 57.4%, and 38.3%, respectively. The median (95% CI) survival time for advanced OCCC patients was 26.80 (22.15–29.84) months, shorter than the 44.45 (42.76–45.24) months of advanced OSC. Taken together, advanced (stage III–IV) OCCC showed a particularly poor prognosis, which should be of concern to researchers as a type of high-risk OCCC.

### COX regression and nomogram

For the analysis of advanced stage OCCC, we employed univariable and multivariable COX regression models to identify prognostic factors influencing survival outcomes in patients with advanced OCCC. 563 cases of stage III/IV were initially identified, and a total of 366 cases were included in the analysis by screening out 184 cases missing CA125 information and 13 cases missing laterality information. The univariate analysis indicated that age, tumor numbers, T-stage, M-stage, laterality, CA125, liver metastases, lymph node-dissection (LN-dissection), surgery, and chemotherapy were significantly associated with CSS (*p* < 0.05). In the further multivariable analysis, age [45–65 y HR (95%CI): 0.603 (0.414–0.878) compared with < 45 y], tumor number [tumor number2 (with two tumors) HR (95%CI): 0.528 (0.338–0.825) compared with the former group (with only one tumor)], T-stage [T3 HR (95%CI): 3.060 (1.894–4.944) compared with T1], bilateral tumor [HR (95%CI): 1.831(1.393–2.406)], liver metastases [HR (95%CI): 2.887(1.783–4.676)], could be defined as independent prognostic factors (*p* < 0.05). Of note, the result supported findings that the performances of surgery [surgery HR (95%CI): 0.212(0.127–0.352) compared with no surgery] and chemotherapy [chemotherapy HR (95%CI): 0.364(0.238–0.556)] were significantly and independently associated to better CSS, while LN-dissection [(LN-dissection HR (95%CI): 0.913(0.673–1.238)] (*p* = 0.558) was not an independent prognostic factor of advanced stage OCCC patients (Table 1). In addition, we applied a LASSO regression algorithm based on features in the univariate Cox regression analyses. The most appropriate tuning parameter λ for LASSO regression was 0.1364; 4–11 variables with nonzero coefficients were suggested in the LASSO analysis (Additional file 1).

**Table 1:** Univariate and multivariate Cox regression of cancer-specific survival among advanced stage OCCC patients

Variable	**N**	Univariate		Multivariate	
		HR (95%CI)	*p*-value	HR (95%CI)	*p*-value
**Age (years)**			**0.005**		**0.038**
<45	48	reference		reference	
45-65	207	0.592(0.410-0.856)	**0.005**	0.603(0.414-0.878)	**0.008**
>65	111	0.841(0.567-1.247)	0.389	0.706(0.469-1.063)	0.095
**Tumor numbers**			**0.008**		**0.008**
1	305	reference			
2	48	0.548(0.353-0.851)	**0.007**	0.528(0.338-0.825)	**0.005**
3	10	0.388(0.124-1.215)	0.104	0.619(0.196-1.949)	0.412
4	3	2.056(0.656-6.445)	0.216	2.445(0.763-7.831)	0.132
**T stage**			**<0.001**		**<0.001**
T1	55	reference		reference	
T2	28	1.638(0.843-3.181)	0.145	1.922(0.973-3.794)	0.060
T3	283	2.955(1.881-4.642)	**<0.001**	3.060(1.894-4.944)	**<0.001**
**M stage**					
M0	270	reference		reference	
M1	96	1.683(1.272-2.228)	**<0.001**	1.243(0.888-1.739)	0.205
**N stage**					
N0	194	reference			
N1	172	0.798(0.617-1.032)	0.085		
**Laterality**					
Unilateral	234	reference		reference	
Bilateral	132	1.994(1.538-2.586)	**<0.001**	1.831(1.393-2.406)	**<0.001**
**CA125**					
Negative	36	reference		reference	
Positive	330	1.742(1.088-2.789)	**0.021**	1.480(0.910-2.406)	0.114
**Liver metastases**					
No	342	reference		reference	
Yes	24	3.663(2.309-5.812)	**<0.001**	2.887(1.783-4.676)	**<0.001**
**Lung metastases**					
No	352	reference			
Yes	14	1.255(0.685-2.301)	0.462		
**Surgery**					
No	23	reference		reference	
Yes	343	0.184(0.115-0.295)	**<0.001**	0.212(0.127-0.352)	**<0.001**
**Chemotherapy**					
No	40	reference		reference	
Yes	326	0.582(0.392-0.864)	**0.007**	0.364(0.238-0.556)	**<0.001**
**LN-dissection**					
No	122	reference		reference	
Yes	244	0.534(0.410-0.697)	**<0.001**	0.913(0.673-1.238)	0.558

**Fig. 3:**
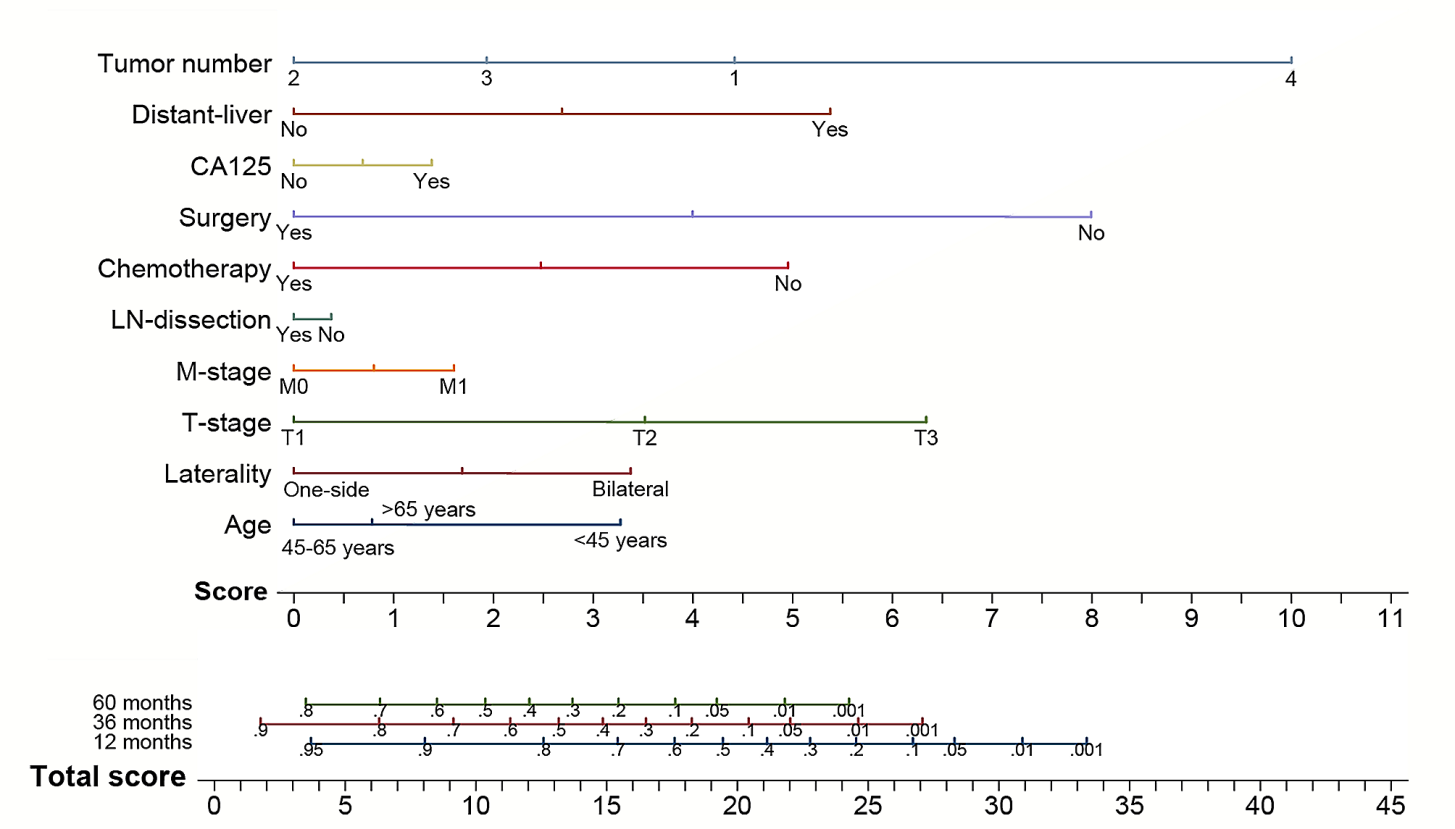
A nomogram to predict 1-, 3-, and 5-year CSS for advanced stage OCCC. CSS, cancer-specific survival

**Fig. 4:**
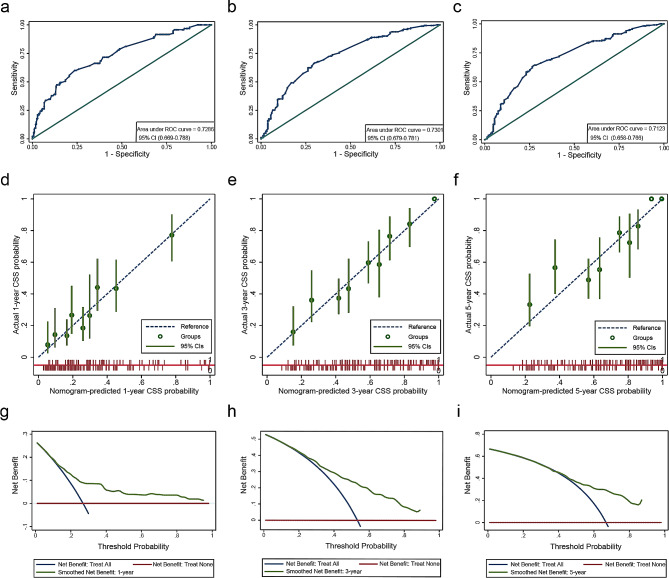
Calibration chart and clinical suitability test of the nomogram. ROC curve of (**a**) 1-year, (**b**) 3- year and (**c**) 5-year CSS for advanced OCCC patients; The calibration curves of (**d**)1- year, (**e**) 3- year and (**f**) 5-year for advanced OCCC patients; DCA curve for advanced OCCC patients CSS at (**g**)1-year, (**h**) 3-year, and (**i**) 5-year. ROC, receiver operating characteristic curve; DCA, Decision Curve Analysis

Based on the results of the preceding analyses, we made a nomogram model of CSS using prognostic factors of advanced OCCC. As illustrated in Fig. 3, 10 variables were included, and each could be assigned to a score ranging from 0 to 10. The 1-, 3-, and 5-year survival rates were calculated using the sum of scores for one patient ranging from 0 to 35. The C-index of the nomogram was 0.7074 (95% CI, 0.6728–0.7428). At the same time, we displayed ROC curves of 1-, 3-, and 5-year CSS to demonstrate the discrimination abilities of the model at critical time points. The area under curves (AUC) were 0.7286, 0.7301, and 0.7123, respectively **(**Fig. 4a-c**)**. Moreover, calibration plots, showing the consistency of the model were generated to represent a favorable prediction for 1-, 3-, and 5-year CSS among advanced OCCC (Stage III-IV) patients **(**Fig. 4d-f**)**. Additionally, decision curve analysis (DCA) at three time points suggested that applying the model would be a good predictor for informing clinical decisions across a wide range of threshold probabilities **(**Fig. 4g-i**)**.

### Survival outcomes of advanced OCCC with different treatments

**Fig. 5:**
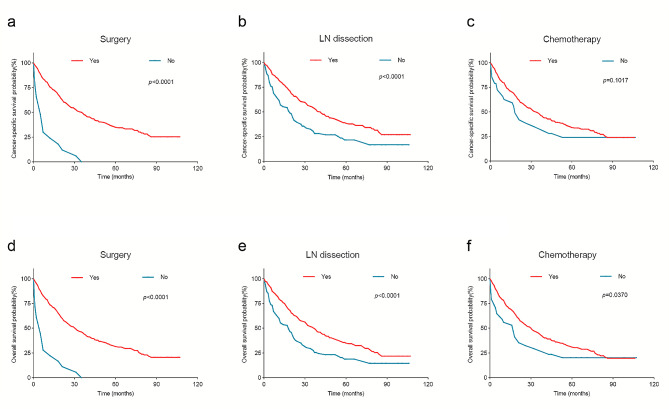
Kaplan–Meier curves for advanced stage OCCC patients according to different treatments. CSS for advanced stage OCCC patients according to (**a**) surgery, (**b**) LN dissection, (**c**) chemotherapy; OS for advanced stage OCCC patients according to (**d**) surgery, (**e**) LN dissection, (**f**) chemotherapy

The median survival time for the 366 advanced OCCC patients was 31 months (95% CI: 24.920–3.080), while the mean survival time was 46.203 months (95% CI: 41.773–50.533). Notably, patients who underwent surgery exhibited significantly higher 1-y CSS rates of 75.1%, compared to those who did not receive surgical intervention, whose rate stood at a mere 20.7% (*p* < 0.0001). Besides, the 3- and 5-year CSS rates for surgery patients were found to be 48.8% and 34.7%, respectively. However, non-surgery patients did not surpass a 3-y threshold in terms of overall survival duration. Furthermore, the 5-y CSS rate of patients with LN dissection vs. without LN dissection was 34.7% vs. 20.8% (*p* < 0.0001). In terms of chemotherapy, the 5-y CSS rate of patients receiving chemotherapy was higher at 34.1% compared to 20.9% for patients not receiving chemotherapy (*p* = 0.1017) **(**Fig. 5a-c**)**. Nevertheless, it is worth noting that less than 5 patients received radiotherapy in this study; therefore, no follow-up analysis regarding its impact could be conducted.

Patients who did and did not receive surgery had 1-y OS rates of 73.1% and 18.2%, respectively (*p* < 0.0001). The 5-y OS rates for patients with lymph node (LN) dissection were higher at 34.9% compared to those without LN dissection, which was only 17.8% (*p* < 0.0001). Furthermore, the administration of chemotherapy resulted in improved 5-y OS rates, with patients receiving chemotherapy achieving a rate of 30.7%, whereas those not receiving chemotherapy had a lower rate of only 17.6% (*p* = 0.0370) **(**Fig. 5d-f**) (**Table 2**)**.

**Table 2:** Five-year survival rate under different treatments

Treatment	*N*	Survival rates			
		5-y CSS rate	*p*-value	5-y OS rate	*p*-value
**Sugery**			< 0.0001		< 0.0001
Yes	343	34.7%		35.1%	
No	23	0%		0%	
**LN-dissection**			< 0.0001		< 0.0001
Yes	244	34.7%		34.9%	
No	122	20.8%		17.8%	
**Chemotherapy**			0.1017		0.0370
Yes	326	34.1%		30.7%	
No	40	20.9%		17.6%	

Furthermore, the selection of the surgical approach holds significant importance. According to the guidelines for diagnosing and treating ovarian cancer, advanced tumors have a direct impact on a patient’s physical status and their likelihood of undergoing surgery. If feasible, surgical treatment should be performed first, including resection of the whole uterus, bilateral fallopian tubes and ovaries, and greater omentum, and the best efforts should be made to achieve optimal surgical debulking. Following neoadjuvant chemotherapy for invasive epithelial ovarian cancer, IDS (Interval Debulking Surgery) should also make the greatest efforts to achieve the maximum tumor reduction effect. After receiving IDS, stage III patients may be considered to use cisplatin for hyperthermic intraperitoneal chemotherapy (HIPEC). That is, the majority of patients should receive PDS, and neoadjuvant chemotherapy is feasible for those whose physical condition is not suitable for immediate surgery or whose possibility of achieving optimal residual disease is low after initial tumor reduction. Regarding ovarian clear cell carcinoma (OCCC), comprehensive surgical staging and postoperative chemotherapy are recommended as initial treatment. Platinum-based chemotherapy is recommended after surgery for patients with advanced stage, and systemic therapy is recommended for patients with stage II-IV. In this study, among 355 advanced patients who underwent surgery, 303 patients were PDS patients, 19 patients received systemic therapy before surgery, and 33 patients received systemic therapy both before and after surgery. However, information regarding HIPEC utilization among these patients was not clearly documented.

### Clinical characteristics of OCCC and OSC patients

The prognostic disadvantage of a subset of OCCC cases compared to OSC remains unclear. Comprehensive comparisons and prognostic assessments are needed to guide future treatments for OCCC. There were 16,767 OSC cases and 3003 OCCC cases included in the current analysis. Demographic and clinical characteristics are presented in Table 3. In terms of the basic information of the patients, in contrast to OSC (8.50%), the proportion (20.58%) of non-white or non-black race in OCCC patients was larger (*p* < 0.001). So, patients with OCCC were more likely to be Asian and younger at diagnosis [< 65 y: 75.26% (OCCC) vs. 52.46% (OSC)] (*p* < 0.001).

**Table 3:** Basic clinicopathological characteristics of ovarian clear cell carcinoma and ovarian serous cancer

Characteristics	Serous carcinoma		Clear cell carcinoma		*p*-value
	*N* (16,767)	*P* (100%)	*N* (3003)	*P* (100%)	
**Race**					**<0.001**
White	13,935	83.11	2228	74.19	
Black	1339	7.99	143	4.76	
Other	1426	8.50	618	20.58	
Unknown	67	0.40	14	0.47	
**Marital status**					**<0.001**
Married	8805	52.51	2086	69.46	
Unmarried	7229	43.12	781	26.01	
Unknown	733	4.37	136	4.53	
**Age**					**<0.001**
<40	558	3.33	147	4.90	
40-64	8237	49.13	2113	70.36	
≥65	7972	47.54	743	24.74	
**Grade**					**<0.001**
I	372	2.22	23	0.77	
II	899	5.36	170	5.66	
III	5048	30.11	928	30.90	
IV	5217	31.11	618	20.58	
Unknown	5231	31.20	1264	42.09	
**T-Stage**					**<0.001**
T1	1109	6.61	1135	37.80	
T2	1302	7.77	243	8.09	
T3	6843	40.81	364	12.12	
Unknown	7513	44.81	1261	41.99	
**N-Stage**					**<0.001**
N0	6463	38.54	1517	50.52	
N1	2833	16.90	227	7.56	
Unknown	7471	44.56	1259	41.92	
**M-Stage**					**<0.001**
M0	6868	40.6	1611	53.65	
M1	2428	14.48	133	4.43	
Unknown	7471	44.56	1259	41.92	
**Surgery performed**					**<0.001**
Yes	14,834	88.47	2924	97.37	
No	1933	11.53	79	2.63	
**Chemotherapy**					**<0.001**
Yes	13,944	83.16	2353	78.35	
No	2823	16.84	650	21.65	
**Distant lymph nodes**					**<0.001**
Positive	488	2.91	24	0.80	
Negative	5598	33.39	965	32.13	
Unknown	10,681	63.70	2014	67.07	
**Lung metastases**					**<0.001**
Yes	771	4.60	45	1.50	
No	14,520	86.60	2678	89.18	
Unknown	1476	8.80	280	9.32	
**Liver metastases**					**<0.001**
Yes	980	5.85	61	2.03	
No	14,328	85.45	2666	88.78	
Unknown	1459	8.70	276	9.19	

As for TNM-stage, OCCC patients were more likely to be diagnosed at T1 (37.80%), N0 (50.52%), and M0 (53.65%) compared to 6.61%, 38.54%, and 40.96% of OSC patients (*p* < 0.001), respectively. Moreover, the proportion (1.50%) of lung metastases in OCCC patients were significantly lower than those in OSC patients (4.60%) (*p* < 0.001). And liver metastases occur in 2.03% of OCCC patients, compared to 5.85% of patients with serous cancer (*p* < 0.001). In terms of treatment, OCCC patients were more likely to undergo surgery but not receive chemotherapy compared with OSC patients (*p* < 0.001). In addition, the differences between the groups remained statistically significant after correction of the significant values according to Bonferroni method. Overall, our results suggested that OCCC was significantly different from OSC in demographic characteristics, tumor characteristics, and treatment.

### Prognostic factors only for OCCC patients

Since the treatment principle of OCCC is based on OSC, we tried to identify OCCC-specific prognostic factors that were different from OSC and are therefore often overlooked in clinical practice. Initially, we extracted data for OCCC and OSC separately. The multivariate Cox regression model of OSC showed that age, grade, TNM-stage, bilateral, CA125, distant lymph node, surgery, and chemotherapy were all significantly associated with CSS (Table 4).

**Fig. 6:**
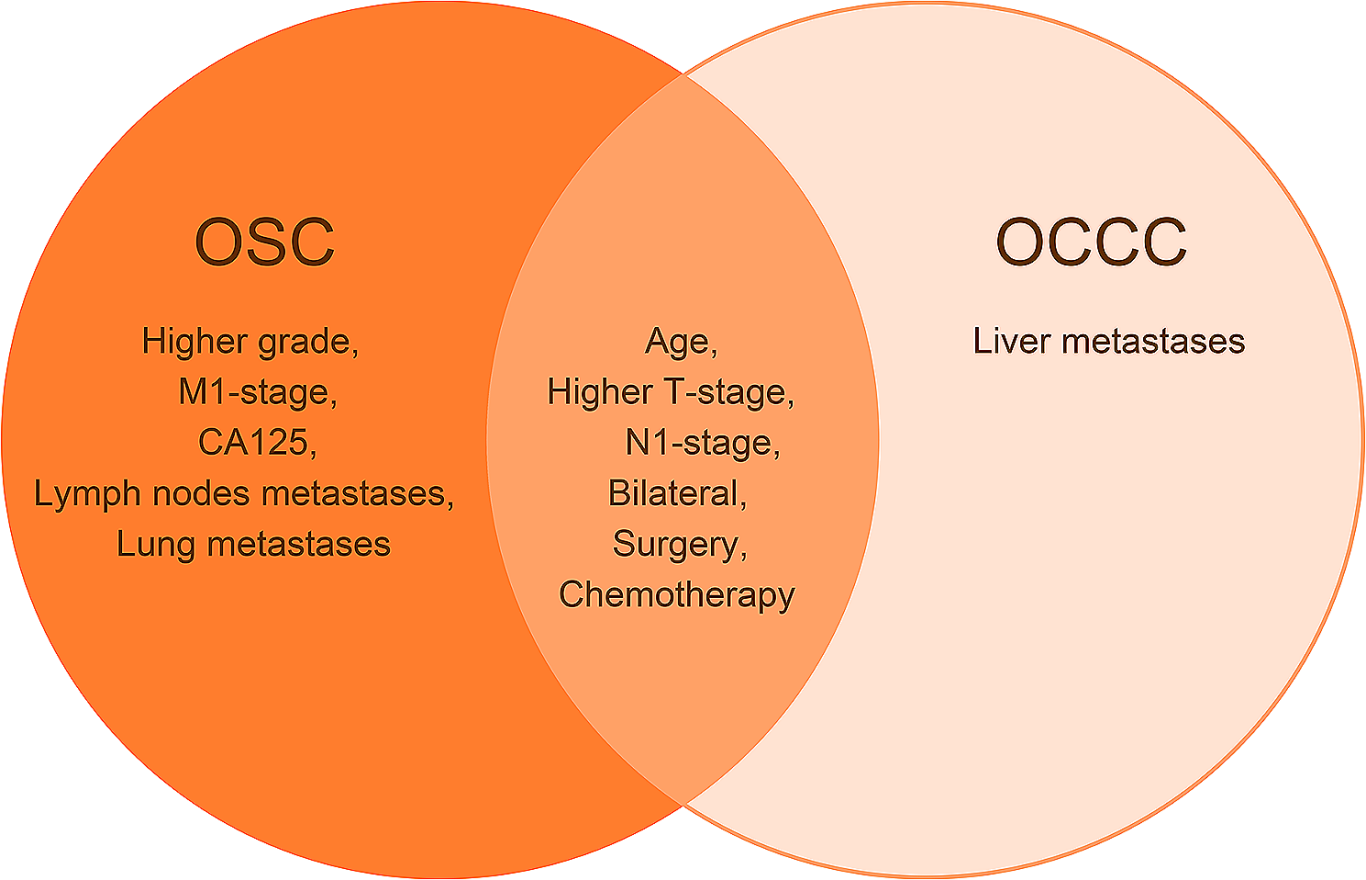
The Venn diagram of prognosis factors of OSC and OCCC. The factors included in the left circle were associated with OSC mortality. The factors included in the right circle were associated with OCCC mortality. Age, higher T-stage, N1- stage, bilateral, surgery, and chemotherapy are both the OSC and OCCC prognostic factors

In the univariate analysis of OCCC, old age (45–65 y), higher TNM-stage, elevated CA125 levels, bilateral involvement, positive lymph node status, and liver metastases were related to a poorer prognosis, while patients who receiving surgery or chemotherapy had better cancer-specific survival time (*p* < 0.05). Multivariate analysis further demonstrated that age, T-stage, N-stage, bilateral, liver metastases, surgery, and chemotherapy showed significant differences **(**Table 5**)**. Based on the above analysis, liver metastasis **(**Fig. 6**)** was a high-risk prognostic factor specific to OCCC, which was different from OSC and was included in our further study.

**Table 4:** Univariate and multivariate Cox regression of CSS analysis for OSC patients

Characteristics	*N*	Univariate		Multivariate	
		HR (95%CI)	*p*-value	HR (95%CI)	*p*-value
**Age**					
<45	1182	reference		reference	
45-65	7613	1.701(1,511-1.916)	**<0.001**	1.596(1.416-1.799)	**<0.001**
>65	7972	3.115(2.770-3.503)	**<0.001**	2.478(2.200-2.790)	**<0.001**
**Grade**					
I	372	reference		reference	
II	899	2.058(1.634-2.591)	**<0.001**	1.772(1.405-2.234)	**<0.001**
III	5048	2.843(2.288-3.534)	**<0.001**	2.380(1.912-2.962)	**<0.001**
IV	5217	2.754(2.215-3.423)	**<0.001**	2.410(1.935-3.001)	**<0.001**
Unknown	5231	NA	NA	NA	NA
**T stage**					
T1	1109	reference		reference	
T2	1302	2.033(1.754-2.357)	**<0.001**	1.942(1.673-2.254)	**<0.001**
T3	6843	3.775(3.333-4.275)	**<0.001**	3.208(2.818-3.651)	**<0.001**
Unknown	7513	NA	NA	NA	NA
**N stage**					
N0	6463	reference		reference	
N1	2833	1.211(1.143-1.283)	**<0.001**	1.064(1.003-1.129)	**0.038**
Unknown	7471	NA	NA	NA	NA
**M stage**					
M0	6868	reference		reference	
M1	2428	1.937(1.828-2.052)	**<0.001**	1.388(1.303-1.480)	**<0.001**
Unknown	7471	NA	NA	NA	NA
**Laterality**					
Unilateral	7144	reference		reference	
Bilateral	9623	1.445(1.378-1.515)	**<0.001**	1.216(1.158-1.277)	**<0.001**
**CA125**					
Negative	873	reference		reference	
Positive	15,894	1.161(1.113-1.211)	**<0.001**	1.099(1.042-1.159)	**0.001**
**Distant lymph nodes**					
No	5598	reference		reference	
Yes	488	1.626(1.374-1.925)	**<0.001**	1.208(1.019-1.432)	**0.030**
Unknown	10,681	NA	NA	NA	NA
**Liver metastases**					
No	14,328	reference			
Yes	980	1.097(0.768-1.567)	0.611		
Unknown	1459	NA	NA		
**Lung metastases**					
No	14,520	reference		reference	
Yes	771	2.037(1.856-2.236)	**<0.001**	1.245(1.126-1.376)	**<0.001**
Unknown	1476	NA	NA	NA	NA
**Surgery**					
No	1933	reference		reference	
Yes	14,834	0.178(0.167-0.188)	**<0.001**	0.242(0.226-0.260)	**<0.001**
**Chemotherapy**					
No	2823	reference		reference	
Yes	13,944	0.629(0.594-0.665)	**<0.001**	0.515(0.485-0.546)	**<0.001**

**Table 5:** Univariate and multivariate Cox regression of CSS analysis for OCCC patients

Characteristics	*N*	Univariate		Multivariate	
		HR (95%CI)	*p*-value	HR (95%CI)	*p*-value
**Age**					
<45	348	reference		reference	
45-65	1914	0.656(0.494-0.873)	**0.004**	0.626(0.470-0.833)	**0.001**
>65	741	1.008(0.748-1.358)	0.959	0.878(0.649-1.187)	0.398
**Grade**					
I	23	reference			
II	170	0.739(0.311-1.757)	0.494		
III	928	1.367(0.610-3.067)	0.448		
IV	618	1.118(0.496-2.524)	0.788		
Unknown	1264	NA	NA		
**T stage**					
T1	1135	reference		reference	
T2	243	2.789(2.198-3.538)	**<0.001**	2.637(2.073-3.353)	**<0.001**
T3	364	7.412(6.176-8.895)	**<0.001**	5.067(4.086-6.284)	**<0.001**
Unknown	1261	NA	NA	NA	NA
**N stage**					
N0	1517	reference		reference	
N1	227	3.414(2.836-4.109)	**<0.001**	1.418(1.152-1.744)	**0.001**
Unknown	1259	NA	NA	NA	NA
**M stage**					
M0	1611	reference		reference	
M1	133	5.134(4.154-6.346)	**<0.001**	1.169(0.886-1.542)	0.271
Unknown	1259	NA	NA	NA	NA
**Laterality**					
Unilateral	2591	reference		reference	
Bilateral	412	3.501(3.002-4.082)	**<0.001**	1.724(1.456-2.043)	**<0.001**
**CA125**					
Negative	581	reference		reference	
Positive	2422	1.174(1.067-1.292)	**<0.001**	0.966(0.857-1.089)	0.570
**Distant lymph nodes**					
No	965	reference		reference	
Yes	24	6.887(3.955-11.993)	**<0.001**	1.174(0.638-2.161)	0.607
Unknown	2014	NA	NA	NA	NA
**Liver metastases**					
No	2666	reference		reference	
Yes	61	8.673(6.437-1.686)	**<0.001**	2.381(1.723-3.290)	**<0.001**
Unknown	276	NA	NA	NA	NA
**Lung metastases**					
No	2678	reference		reference	
Yes	45	4.156(2.882-5.994)	**<0.001**	1.281(0.845-1.942)	0.244
Unknown	280	NA	NA	NA	NA
**Surgery**					
No	79	reference		reference	
Yes	2924	0.068(0.053-0.088)	**<0.001**	0.134(0.101-0.179)	**<0.001**
**Chemotherapy**					
No	650	reference		reference	
Yes	2353	0.792(0.676-0.927)	**0.004**	0.650(0.553-0.766)	**<0.001**

### Risk factors for developing liver metastases

Early detection and timely intervention play a crucial role in determining the prognosis of cancer. In the case of OCCC, liver metastases, which serve as a crucial prognostic factor, should be given significant attention in clinical practice. Subsequently, we extracted data from 61 OCCC cases with liver metastases to explore the associated risk factors and raise awareness for future clinical endeavors. The logistic regression results presented in Table 6 demonstrate that, according to univariate analysis, T3-stage, higher N-stage, bilateral involvement, distant lymph nodes, and lung metastases are all significantly associated with an increased risk of liver metastases. However, after conducting multivariate analysis to control for confounding factors, it was found that T3-stage, positive distant lymph nodes, and lung metastases remained as independent risk factors associated with the development of liver metastases. (*p* < 0.05).

### Survival outcomes with different treatments

**Fig. 7:**
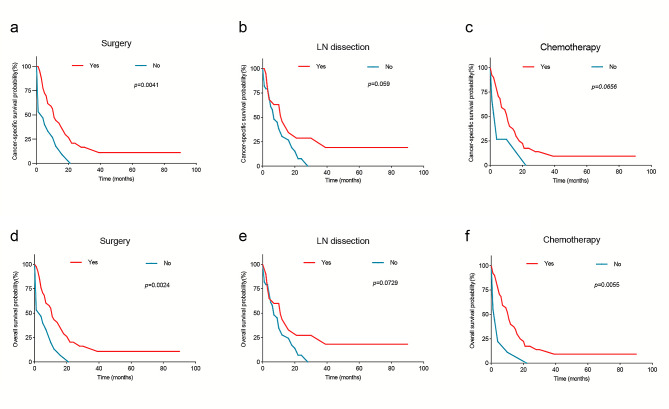
Kaplan–Meier curves for OCCC patients with liver metastases according to different treatments. CSS for OCCC patients with liver metastases according to (**a**) surgery, (**b**) LN dissection, (**c**) chemotherapy; OS for OCCC patients with liver metastases according to (**d**) surgery, (**e**) LN dissection, (**f**) chemotherapy

Among the 61 patients with liver metastases in OCCC, 45 patients received surgical intervention, and the 1- and 3-y CSS rates were 44.4% and 11.1%. Conversely, among the remaining 16 patients who did not undergo surgery, none survived beyond a period of 20 months, and the 1-y CSS rate was 16.7%, significantly lower than that of patients who underwent surgery (*p* = 0.0041) **(**Fig. 7a**).** Of the 45 patients with liver metastasis who underwent surgical treatment, 30 patients received PDS, 2 patients received systemic therapy before surgery, and 9 patients received systemic therapy both before and after surgery. Regarding lymph node dissection, the 1-y CSS rates for patients who underwent or did not undergo this procedure were recorded as being 45.9% and 31.9%, respectively **(**Fig. 7b**)** (*p* = 0.059). Among the 10 patients who did not receive chemotherapy, only one survived beyond 1 year, while the 1-y CSS rate of OCCC patients who receive chemotherapy was 39.8%. Moreover, the median survival time of the former group (2 months, 95% CI: 0–4.467) was significantly shorter than the latter (11 months, 95% CI: 8.584–13.416) (*p* < 0.05). But chemotherapy did not have a statistically significant effect on CSS of patients with liver metastases **(***p* = 0.0656) **(**Fig. 7c**)**. However, only one of the 61 patients with liver metastases received radiotherapy.

Furthermore, surgical intervention and chemotherapy exhibited notable impacts on the overall survival rates and duration of survival in patients with liver metastases from OCCC, as illustrated in Fig. 7d-f.

**Table 6:** Univariate and multivariate logistic regression of liver metastases for OCCC patients

Characteristics	*N*	Univariate		Multivariate	
		HR (95%CI)	*p*- value	HR (95%CI)	*p*-value
**Age**					
<45	1	reference			
45-65	40	2.665(0.363-19.548)	0.335		
>65	20	3.759(0.500-28.267)	0.198		
**Grade**					
I	1	reference			
II	17	NA	0.998		
III	7	0.200(0.027-1.466)	0.113		
IV	36	0.645(0.360-1.157)	0.142		
Unknown	0	NA	NA		
**T stage**					
T1	2	reference		reference	
T2	2	4.761(0.667-33.964)	0.120	3.652(0.494-26.977)	0.204
T3	28	47.777(11.323-201.603)	**<0.001**	37.392(8.764-159.540)	**<0.001**
Unknown	29	NA	NA	NA	NA
**N stage**					
N0	17	reference		reference	
N1	16	6.696(3.332-13.454)	**<0.001**	1.664(0.751-3.687)	0.210
Unknown	28	NA	NA	NA	NA
**M stage**					
M0	33	reference			
M1	28	NA	0.984		
Unknown	0	NA	NA		
**Laterality**					
Unilateral	35	reference		reference	
Bilateral	26	5.325(3.173-8.936)	**<0.001**	1.490(0.789-2.812)	0.219
**CA125**					
Negative	45	reference			
Positive	16	1.381(0.939-2.032)	0.101		
**Distant lymph nodes**					
No	18	reference		reference	
Yes	11	39.832(15.519-102.238)	**<0.001**	32.730(11.727-91.345)	**<0.001**
Unknown	32	NA	NA	NA	NA
**Lung metastases**					
No	43	reference		reference	
Yes	15	31.696(15.861-63.342)	**<0.001**	17.399(7.631-39.673)	**<0.001**
Unknown	3	NA	NA	NA	NA

### Detection of GPC3 protein expression in OCCC by IHC

**Fig. 8:**
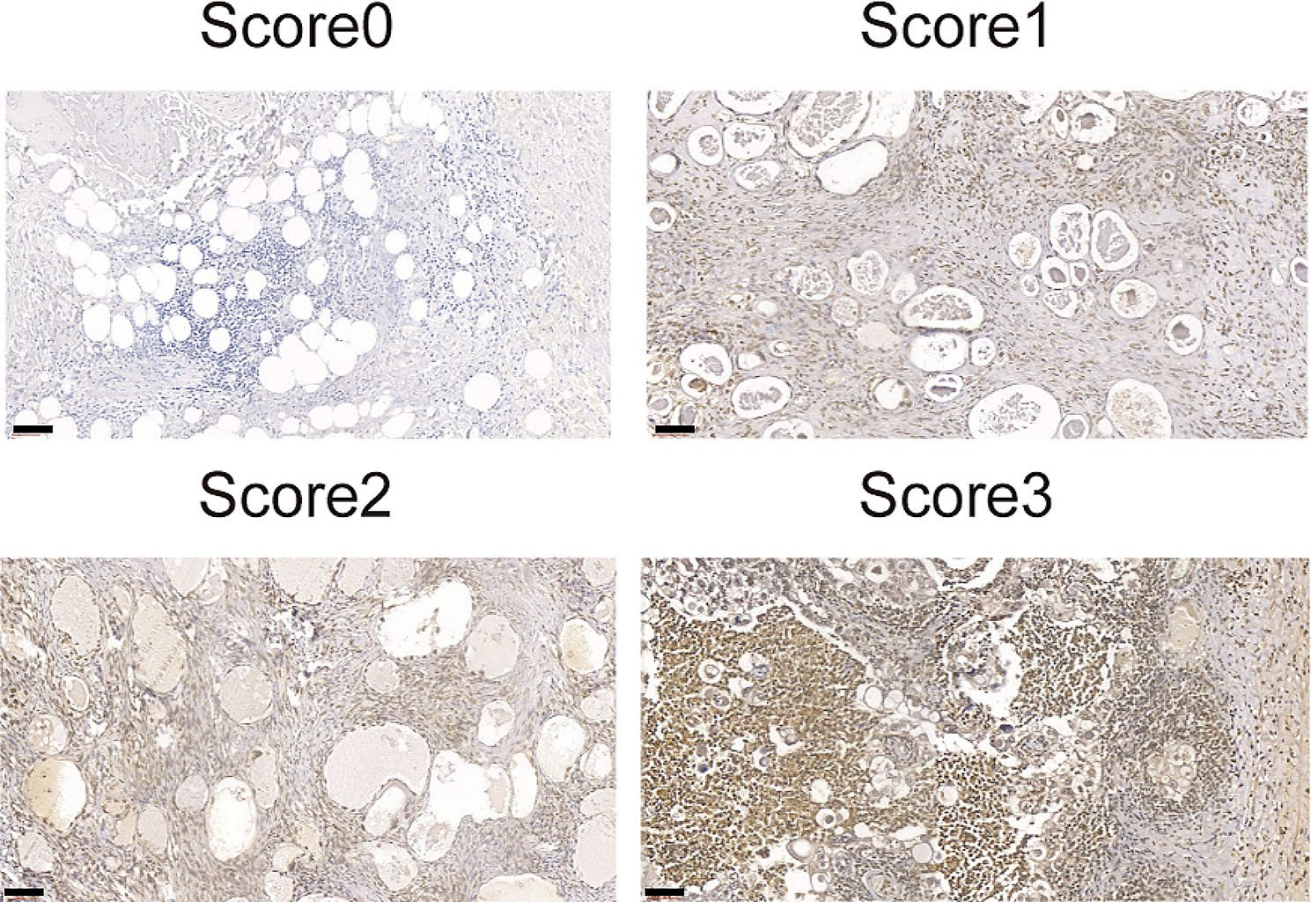
Representative images of the expression of GPC3 protein in OCCC tissues by IHC. Score 0 indicated that none or little cells express GPC3 (Negative control: GPC3-negative normal tissues); Score 1 indicated that more than 25% of tumor cells have weak expression of GPC3; Score 2 indicated more than 50% of tumor cells have weak expression or more than 25% of tumor cells have moderate expression of GPC3 protein; Score 3 indicated more than 75% of tumor cells have moderate expression or more than 50% of tumor cells have strong expression of GPC3. The scale bar in the bottom left corner of each picture represents 60 µM

We conducted an analysis on various conventional treatments for ovarian clear cell carcinoma (OCCC). In general, aggressive surgery and chemotherapy had a positive impact on survival outcomes for both types of high-risk OCCC. However, the overall prognosis of such patients remained unfavorable. There is an urgent need to find new treatments based on the molecular properties of OCCC. GPC3 is a member of the GPC family, which is widely conserved across species and plays an important role in biological processes. It can be used as a co-receptor for a variety of signaling molecules to regulate cell growth, motility and differentiation. Nowadays, it is being explored as a hot potential candidate for HCC, OCCC and other solid tumors immunotherapy. We used immunohistochemistry to detect the expression frequency of glypican-3 (GPC3) in OCCC, which is a well-recognized diagnostic marker of the hepatocellular carcinoma and highly heterogeneous in other cancers such as OCCC [19, 20], so as to provide a clear clinical basis for future drug development. Thirteen OCCC tissue sections were all obtained from surgically confirmed cases in Shanghai First Maternity and Infant Health Hospital. Importantly, GPC3 expression was detected in all 13 tissue sections, and GPC3 staining pattern was predominantly cytoplasmic with concomitant membranes **(**Fig. 8**)**. Among 13 OCCC tissues, there were 5 cases of score1, 3 cases of score2, and 3 cases of score3. Intertumoral heterogeneity also showed that nuclear GPC3 expression tested positive in part of tissue block area.

## Discussion

This study compared the clinical and prognostic characteristics of OCCC and OSC. On this basis, we focused on analyzing the risk factors and prognostic outcomes of two high-risk types of OCCC, especially from several therapeutic perspectives. We further established and validated a prognostic nomogram for stage III–IV OCCC. This model can accurately identify advanced OCCC patients who would benefit from several treatments and assist in making individual recommendations. Due to the relatively low incidence of this tumor, there have been limited previous reports and clinical studies discussing OCCC. The ultimate purpose of this study is to increase clinical awareness of high-risk OCCC patients. Our results help clinicians assess the prognosis of high-risk OCCC patients and manage them.

Unlike OSC, several studies have confirmed that OCCC can be easily diagnosed at an early stage due to its manifestation as a large pelvic mass. However, OCCC patients exhibit a highly heterogeneous prognosis [21]. Endometriosis is reported to be an independent risk factor for OCCC and ovarian endometriotic epithelial cells are considered as the cellular source of OCCC [22, 23]. Even so, the mechanism of tumorigenesis has not been fully elucidated. However, we can confirm that it has distinct histopathological, and molecular characteristics compared with other histologic subtypes of epithelial ovarian cancer [4, 24, 25]. Previous studies have established that the prognosis of advanced OCCC is far from satisfactory, even worse than that of advanced OSC [26]. This aligns with our findings, which demonstrate that survival outcomes for late-stage OCCC patients are even poorer compared to those in early-stage. Hence, it is crucial to prioritize attention towards this type of high-risk OCCC. In this study, the univariable analysis indicated that older age, lower differentiation, positive CA125, liver metastases, and no-treatment affected the prognosis of advanced OCCC. In the aspect of clinical characteristics, multivariable analysis further identified age (45–65 y), T3-stage, bilateral, and liver metastases as independent prognostic factors. It has been reported that OCCC is usually diagnosed in younger patients (average age: 56 y) [27]. The proportion of patients under the age of 50 is also on the rise. It is not surprising that a younger patient age is a factor for favorable prognosis in OCCC. Our study confirms that advanced OCCC tends to have a poorer prognosis in older individuals, aligning with the notion that aging represents the greatest risk factor for cancers [28]. Only a few reports have previously demonstrated that OCCC occurs mostly in unilateral ovaries and rarely in bilateral ovaries [29, 30]. Our findings indicated that bilateral occurrence was also an independent factor for poor prognosis in advanced OCCC. It can be inferred that most OCCC is diagnosed at an early stage and has not progressed to become bilateral, while the bilateral occurrence of advanced OCCC is associated with direct spread and invasive development, which are also important factors affecting the prognosis of tumor patients. The prognostic factors of OCCC have been extensively investigated, with limited attention given to liver metastases [31, 32]. The reason may be that the incidence of intraperitoneal dissemination is higher than that of hematogenous metastasis in epithelial ovarian cancer. Our study reveals that liver metastasis is an independent prognostic factor for advanced OCCC, providing insight into the poorer survival outcomes observed in these patients. Furthermore, a nomogram was constructed to assess the 1-, 3-, and 5-year CSS of stage III–IV OCCC based on the identified prognostic factors. Favorable discrimination and calibration were observed from C-index, calibration curves, and DCA curves, indicating excellent performance of the nomogram.

Another notable point is that the clinical characteristics of OCCC and OSC were widely different. These findings imply that there may be challenges in fully adhering to the therapeutic approach for serous carcinoma. Importantly, analysis of prognostic factors for these two epithelial tumors revealed liver metastases as an independent prognostic factor specifically in OCCC, distinguishing it from OSC. However, existing literature on OCCC with liver metastases remains limited or predominantly consists of case reports. Therefore, we hypothesized that liver metastases may often go unnoticed in clinical practice and become another high-risk factor for OCCC. The results of logistic analysis implied that patients with late T-stage, distant lymph nodes metastases, and lung metastases at the first treatment were more susceptible to developing liver metastases. The process of tumor metastasis is very complex, mainly including destruction of stroma, migration, and infiltration of blood vessels or lymphatics. Patients with advanced ovarian cancer are prone to hematogenous metastasis and lymph node invasion, and the common organs for distant metastasis are the lungs, bone, brain, and liver [33, 34]. Our results showed that liver metastases commonly coexisted with lung metastases or promoted its progression, as they were both caused by hematogenous spread. It is not difficult to understand that late T-stage is a risk factor for liver metastases, since tumor cells with high infiltration ability and strong destructive power to the external matrix are more likely to invade blood vessels, thereby causing distant metastasis. Accordingly, we recommend that OCCC patients with lymph node metastases or lung metastases should conduct imaging assessment to detect early liver metastases.

The initial treatment of OCCC consisted of comprehensive surgical staging and postoperative chemotherapy [35–37]. The probability of receiving standard treatment was reduced by 50% in elderly patients compared to their younger counterparts. But elderly patients can also benefit from standard treatment [38]. However, surgical/chemo/radio treatments could seriously affect women’s survival and quality of life, which may also be important factors in whether treatments are initiated [39, 40]. Our study also performed survival analysis for these two high-risk types of OCCC from several therapeutic perspectives. Previous research has indicated that despite most patients receiving adjuvant chemotherapy based on the regimen used for serous carcinomas, OCCC is prone to develop resistance to first-line classic platinum-based chemotherapy drugs [41], which may also be related to the low prevalence of BRCA1/2 mutations in OCCC [42]. From the advanced OCCC aspect, the multivariate Cox regression demonstrated chemotherapy is an independent prognostic factor for CSS in patients. And the K-M method indicated a better overall survival outcome for patients with chemotherapy compared with those who did not, although the difference was small. Thus, chemotherapy was the best choice for women with advanced OCCC to prevent recurrence. As for OCCC patients with liver metastases, chemotherapy also showed significant improvement for poor median survival. These results align with the discoveries made by Jing-He Lang [43]. However, it should be noted that patients with liver metastases who received chemotherapy had better cancer-specific survival outcomes compared to those who did not, although the difference was not statistically significant. It is understandable that chemotherapy has acute or chronic toxic side effects, and the physical condition of patients with distant metastasis is not easy to withstand the toxicity of chemotherapy [44]. Based on the evidence shown above, our study underscores the critical importance of chemotherapy in enhancing prognosis for high-risk OCCC patients. However, careful consideration should be given to chemotherapy for patients with liver metastases. And researchers should focus on fully utilizing technology in tissue proteomics, such as mass spectrometry and protein microarrays to dissect mechanisms of resistance and developing new chemotherapy strategies [45], given that advanced stage OCCC has a lower response rate to the first and the second line chemotherapy when compared to OSC.

At present, surgery remains primary therapeutic approach for OCCC. Our results showed the overall survival rate of advanced OCCC patients who underwent surgery to be much higher than that of patients who did not. This observation holds true even in cases of OCCC with liver metastases. Moreover, surgical approach, the thoroughness of the operation, and preservation of reproductive function are also key factors for the prognosis of patients after surgery [46]. However, reports on the effect of surgical thoroughness on survival in OC patients have been inconsistent, mainly in the possibility of surgical complications caused by lymph node dissection [47–50], but two retrospective studies found that lymphadenectomy was significantly associated with longer DFS [51, 52]. Based on the previous findings, our study analyzed the effect of lymph node dissection on the survival of advanced stage patients and patients with liver metastases. As expected, the implementation of systematic lymphatic dissection emerges as a relatively favorable approach to improve survival rates among patients in advanced stages. Nevertheless, the outcomes revealed no robust and statistically significant association between lymphatic dissection and better OS or CSS in patients with liver metastases. Consequently, it is advisable that we should comprehensively assess the patients’ disease characteristics and risk factors before proceeding with lymphatic dissection.

At present, targeted therapies and immune checkpoint inhibitors have been developed for various cancers, including EOC. PARP inhibitors and the anti-VEGF-A antibodies are two types of approved and most effective targeted drugs for OC at present. Several inhibitors of the PI3K pathway have been developed in preclinical investigations and early clinical trials [53]. Furthermore, the combination of FDA-approved PARP inhibitors with immunotherapies such as anti- PD-1/PD-L1 is based on the hypothesis that PARP inhibitors can restore anti-tumor immune response, particularly in HR deficiency cells, thereby synergizing with ICI to enhance the anti-tumor effect [54]. Therefore, in the absence of standard treatment for OCCC and with immunotherapy demonstrating initial success in other tumors, there is an urgent imperative to identify tumour-specific antigens of OCCC. While there is still little research on targeted therapies for OCCC, a team from Zhongshan Hospital in China recently revealed a case of a patient with advanced Human epidermal growth factor receptor 2 (HER2) -positive OCCC, who was treated with pyrotinib and achieved a PFS of 28 months [55]. This study was based on the fact that the HER2 is positively expressed in 14–45.6% of patients with OCCC [56, 57]. However, this research was limited to one case and more clinical trials and large cohort studies are needed to confirm the efficacy and safety. As we know, the gene expression profile of OCCC is similarly to renal clear cell carcinoma (RCC) and hepatocellular carcinoma (HCC), and GPC3 is an established diagnostic marker for HCC [58–60]. This similarity of pathological and genetic profiles provides some basis and ideas for OCCC cell therapy and targeted immunotherapy. GPC3 is a membrane-bound heparan sulfate proteoglycan, which is expressed during embryonal development and epigenetically silenced in most adult organs and may overexpress during malignant transformation [61]. In recent years, GPC3 has become an interesting candidate for targeted immunotherapy. Antibody-based therapies for HCC, lung squamous cell carcinoma (LSCC) and other GPC3-expressed solid tumors are being investigated in preclinical and clinical studies, and several clinical trials using GPC3-targeted CAR-T cells are underway as well. Clinical trials of drugs targeting GPC3 in OCCC are also currently underway. A phase II trial even reported two patients with chemotherapy-refractory OCCC who achieved a significant clinical response after receiving treatment with GPC3 peptide vaccine. Another phase II trial was conducted to evaluate the effect of GPC3 peptide vaccine against refractory OCCC patients. There were 32 patients included and their results suggest that GPC3 peptide vaccinations may hold a significant impact to prolong survival of patients with refractory OCCC, allowing them to maintain quality of life with no serious toxicities. These interesting findings about GPC3 have not been seen in other types of ovarian epithelial tumors [62–64]. For researchers, we need to accumulate a large amount of clinical data to support the broad clinical applicability of drugs targeting GPC3 in OCCC. A study in Japan showed that GPC3 expression was observed in 44% of clear cell adenocarcinomas, whereas it was rarely observed in other histological subtypes. And overexpression of GPC3 may be related to the development and aggressive behavior of ovarian clear cell adenocarcinoma [65]. One research from Switzerland showed GPC3 was expressed in a total of 17.9% of ovarian carcinomas and was strongly associated with the clear-cell histotype (*P* = 0.0001) [66]. Another research retrospectively assessed the expression frequency and distribution of GPC3 in 316 OCCC specimens from Canadian patients and expression of GPC3 in 58% of cases and high interobserver reproducibility [67]. There is still a lack of such research in China. Based on the above literature accumulation, we preliminarily explored the antigen coverage of GPC3 in OCCC. In our study, GPC3 expression was detected in tumor tissue of all 13 OCCC patients. We will continue to collect more samples for testing and verification to lay a research foundation for the further exploitation of drug targets.

We constructed a nomogram for advanced OCCC and identified liver metastases as another high-risk subtype. Additionally, we investigated the risk factors for developing liver metastases. Besides, the immunohistochemical identification of GPC3 provides a certain experimental basis for molecular targeted therapy of OCCC in the future. However, one limitation of this study was inadequate information available in the SEER database regarding radiotherapy. The second flaw was that the limited OCCC sample size prevented a full validation of the results. Other major limitations of our study include the lack of data on precise information on the extent of lesion invasion and detailed surgical information.We are currently collecting additional patient data to incorporate this information and assess tumor-associated target antigen expression, aiming to establish a clinical foundation for future oncology treatments.

## Conclusions

We demonstrated that advanced OCCC exhibited even poorer survival outcomes than advanced OSC. The nomogram, based on SEER database, can be utilized in clinical practice to evaluate and predict the prognosis of high-risk patients with OCCC. Liver metastases has profound prognostic value in both overall and advanced OCCC. Our study suggests that OCCC patients should undergo imaging evaluation to detect early liver metastases, and patients with poorly differentiated tumors and lymph node or lung metastases should be more vigilant for the possibility of liver metastases. The impact of chemotherapy on the CSS is not statistically significant for patients with liver metastases; however, surgery has shown improvements in CSS of patients with advanced OCCC and liver metastases. Additionally, the decision to perform lymph node dissection requires a comprehensive assessment of the patient’s physical condition. Considering the limitations of current treatments, it is crucial to develop more effective drug targets.

### Methods

#### Data source and ethics compliance

The Surveillance, Epidemiology, and End Results Program (SEER) online database, encompassing approximately 34.6% of the US population was used to obtain ovarian cancer statistical data for further analyses. SEER*Stat Software 8.3.6 was used to extract the ovarian cancer dataset in order to obtain raw pathological information and clinical information. This study did not require ethics committee approval. And it was performed in accordance with the Helsinki Declaration of 1964, and its later amendments.

### Study population

According to the primary site code ICD-O-3 (International Classification of Diseases for Oncology-3)/WHO 2008 restricted to ‘ovary’, all OCCC (8310/3, 8313/3) and OSC (8441/3–8442/3, 8460/3–8462/3) patients diagnosed between 2009 and 2018 were identified and included in our study. The following raw data were extracted from the SEER database for each case: patient ID, year of diagnosis, age at diagnosis, race, Grade (2017), AJCC-TNM, CA-125 Pretreatment Interpretation Recode, distant lymph nodes metastases, total number of tumors for patient, SEER Combined Mets at DX-lung/liver (2010+), laterality, reason of no cancer-directed surgery, chemotherapy recode, radiotherapy recode, survival months, vital status recode (study cutoff used). In this study, we collected and categorized the cohort based on age, race, marital status, laterality, Grade, TNM (Tumor, Node and Metastasis) staging, Stage, tumor number, CA125, distant metastases (lung, liver), surgery treatment, chemotherapy, cause-specific death classification, and survival data. Cases of patients lacking race or survival data or therapy information and those with unknown diagnostic confirmation or distant metastasis or stage were excluded. To construct a prognostic model of advanced OCCC, we only included patients with stage III–IV OCCC; patients with missing information on CA125 and laterality were further excluded. As for analyses regarding OCCC with liver metastases, OCCC cases were collected while excluding patients without liver metastases or those with unknown liver metastases.

### Clinical specimens and IHC staining

For all 13 specimens, informed consent was obtained, along with permission of the Medical Ethics Committee of Shanghai First Maternity and Infant Hospital. The tissue was fixed in formalin and subsequently embedded in paraffin for histological analysis. A primary antibody against GPC3 (GPC3-Fc- Biotin) (1:150) was used overnight at 4 °C. Slides were then incubated for another 1 h using secondary antibody S-HRP (1:500) (Abcam, USA). The complex was detected and visualized using DAB complex (#PK-8501, Vector Lab, USA). Furthermore, hematoxylin was used to counterstain the nuclei. Sections were examined under a microscope.

### Statistical analyses

The distributions of various characteristics between the OCCC and OSC groups were compared using a chi-squared (χ2) test. Univariate and multivariate analyses were conducted using the Cox proportional hazards model. OS and CSS were primarily used in the prognostic analysis. OS represents the time from diagnosis to death for any cause or to the last contact. CSS is defined as the time from diagnosis to death owing to OCCC. Kaplan-Meier (KM) method and the Log-rank test were employed to display all the survival curves and compare the statistical differences of various groups. Univariate and multivariate logistic regression analyses were performed to select risk factors for liver metastases in OCCC patients. A p-value < 0.05 was regarded as statistically significant in all analyses.

The prognostic nomogram of advanced OCCC was generated by StataSE 15 according to the outcomes of multivariable analysis. Harrel’s concordance index (C-index), ROC curve, calibration curve, and DCA curve were used to measure the performance of this nomogram. All other statistical analyses were completed using R software v.4.2.1, SPSS v.26.0 and GraphPad Prism v.8.0.

### Electronic supplementary material

Below is the link to the electronic supplementary material.

Additional file 1.

Additional file1.docx.

The results of LASSO regression analysis for advanced OCCC.

(a) Selection of largest λ with an average error within one standard deviation. The partial likelihood binomial deviance is plotted vs. log (λ). (b) LASSO coefficient profiles for clinical features, each coefficient profile plot is produced vs. log (λ) sequence.

Supplementary Material 1

## Data Availability

All the primary data were acquired from the SEER database (https://seer.cancer.gov/seerstat/.).

## Abbreviations

OCCC: Ovarian clear cell carcinoma
OSC: serous ovarian cancer
OC: ovarian cancer
SEER: the Surveillance, Epidemiology, and End Results
CSS: Cancer-specific survival
OS: overall survival
ROC: receiver operating characteristic
EOC: Epithelial ovarian cancer
HGSC: high-grade serous carcinoma
GPC3: glypican-3
HER2: Human epidermal growth factor receptor 2
HCC: hepatocellular carcinoma
