# Supplementary material for: Exploration and prognostic analysis of two types of high-risk ovarian cancers: clear cell vs. serous carcinoma: a population-based study
Source: J Ovarian Res. 2024 Jun 1;17:119. doi: 10.1186/s13048-024-01435-y (PMC11143660; doi:10.1186/s13048-024-01435-y)
Supplement: Supplementary file 1 — Supplementary Material 1 [file 13048_2024_1435_MOESM1_ESM.docx]

a.


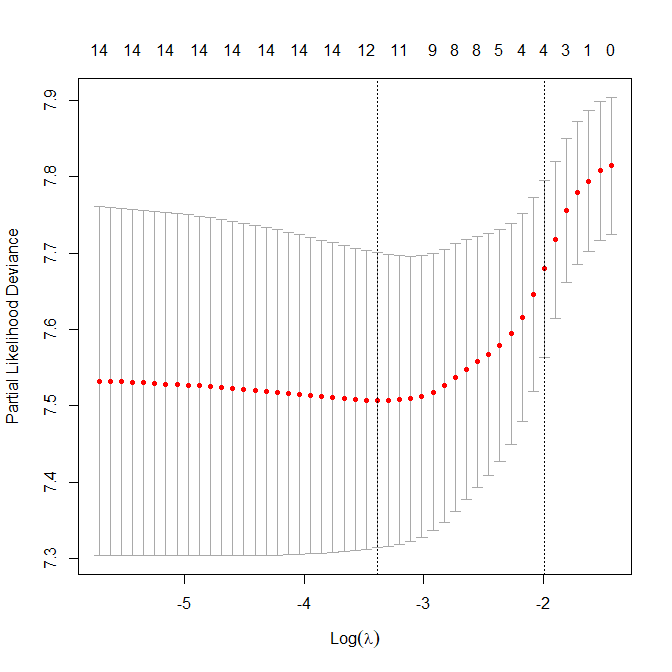


b


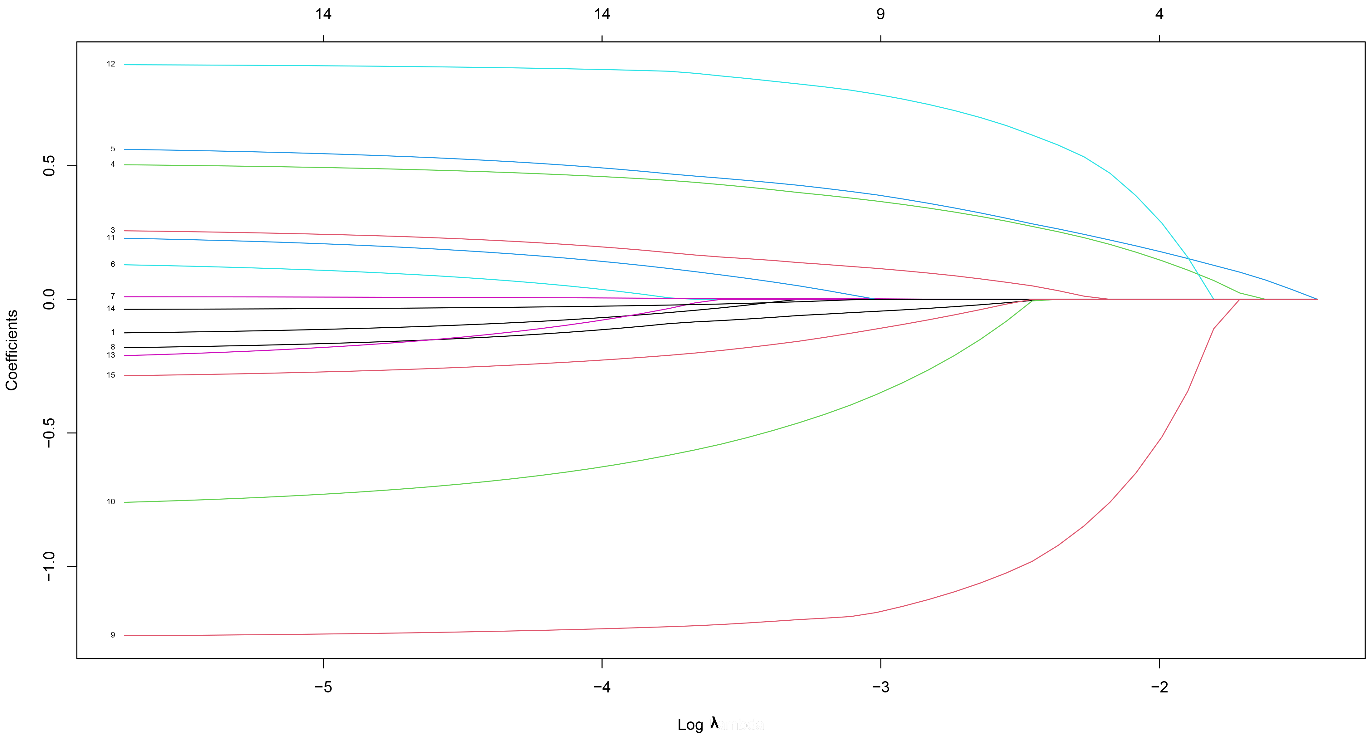


Figure legends

a. Selection of largest λ with an average error within one standard deviation. The partial likelihood binomial deviance is plotted vs log (λ). b. LASSO coefficient profiles for clinical features, each coefficient profile plot is produced vs log (λ) sequence.
